# Fabrication of Planar Microelectrode Array Using Laser-Patterned ITO and SU-8

**DOI:** 10.3390/mi12111347

**Published:** 2021-10-31

**Authors:** Hee Soo Jeong, Seoyoung Hwang, Kyou Sik Min, Sang Beom Jun

**Affiliations:** 1 Department of Electronic and Electrical Engineering, Ewha Womans University, Seoul 03760, Korea; bluehs5@hanmail.net (H.S.J.); qazwsx711@naver.com (S.H.); 2 Graduate Program in Smart Factory, Ewha Womans University, Seoul 03760, Korea; 3 TODOC Co., Ltd., Seoul 08394, Korea; ceo@to-doc.com; 4 Department of Brain and Cognitive Sciences, Ewha Womans University, Seoul 03760, Korea

**Keywords:** microelectrode array, laser, photolithography, SU-8, iridium oxide

## Abstract

For several decades, microelectrode array (MEA) has been a powerful tool for in vitro neural electrophysiology because it provides a unique approach for monitoring the activity of a number of neurons over time. Due to the various applications of MEAs with different types of cells and tissues, there is an increasing need to customize the electrode designs. However, the fabrication of conventional MEAs requires several microfabrication procedures of deposition, etching, and photolithography. In this study, we proposed a simple fabrication method with a laser-patterned indium tin oxide (ITO) conductor and SU-8 photoresist insulation. Unlike in a conventional metal patterning process, only the outlines of ITO conductors are ablated by laser without removing background ITO. Insulation is achieved simply via SU-8 photolithography. The electrode sites are electroplated with iridium oxide (IrO_X_) to improve the electrochemical properties. The fabricated MEAs are electrochemically characterized and the stability of insulation is also confirmed by impedance monitoring for three weeks. Dissociated neurons of rat hippocampi are cultured on MEAs to verify the biocompatibility and the capacity for extracellular neural recording. The electrochemical and electrophysiological results with the fabricated MEAs are similar to those from conventional SiN_X_-insulated MEAs. Therefore, the proposed MEA with laser-patterned ITO and SU-8 is cost-effective and equivalently feasible compared with the conventional MEAs fabricated using thin-film microfabrication techniques.

## 1. Introduction

Planar-type microelectrode arrays (MEAs) have become a useful tool for in vitro studies with electrogenic cells such as neurons, cardiac cells, or muscle cells [1,2,3,4]. Since MEAs enable simultaneous multichannel recording from several tens of individual cells in cultures or slices, the researchers have applied the MEA platform for investigation of signaling in neural networks or high-throughput drug screening [1,5,6,7,8,9,10]. Depending on the research purposes, various technologies have been utilized for the fabrication of MEAs including different designs and materials, surface patterning, microfluidics, and so forth [11,12,13,14,15,16,17,18].

Despite the various applications, MEAs have a common basic structure comprising a transparent substrate, conductive metal patterns, and an insulation layer. All the materials are required to be biocompatible and stable in aqueous culture conditions with various chemicals and proteins. Therefore, gold, platinum, titanium nitride, and indium tin oxide (ITO) are typical conductor materials due to their biocompatibility and stability [19,20,21]. Various insulating materials have been utilized including SU-8, polyimide, polydimethylsiloxane (PDMS), silicon oxide (SiO_2_), silicon nitride (Si_3_N_4_), parylene and so forth [18,22,23,24,25]. Even though those materials are known to be biocompatible and chemically stable, MEAs can provide durability only for a limited number of reuses. It is well-known that long-term exposure to a culture medium and recycling cause several stability problems such as delamination between the layers, cracking of the deposited insulators, or destruction of electroplated electrode sites [26,27,28,29].

In addition to the limited durability of MEAs, in practice, a large-scale experiment with MEAs also burdens researchers with a high cost of MEAs. Typically, MEAs are fabricated using thin-film microfabrication processes including metal deposition, chemical vapor deposition of the insulator, photolithography, and dry or wet etching. Even though it enables precise fabrication of microelectrode arrays, it requires the use of expensive microfabrication equipment and skilled operators, which increases the fabrication cost. In addition, since the microfabrication process is performed based on the predefined photomasks, the change of the MEA design requires additional costs for the production of new photomasks.

In this study, we proposed a simple MEA fabrication method with indium tin oxide (ITO) and an SU-8 photoresist for conductor and insulator materials, respectively. ITO conductor patterns are formed by simple but precise laser ablation. Formation of SU-8 insulation is also achieved via a single photolithography process without the etching process. Compared with the conventional MEA fabrication methods using thin-film microfabrication processes, the proposed method has several advantages. First, conductor patterns can be easily changed using laser ablation without photomask production. Second, the fabrication cost could be reduced because the proposed method includes only a single photolithography process for SU-8 patterning without photolithography for conductor patterning and etching processes. Further, if the insulation layer is also patterned by laser, an MEA can be produced without any conventional microfabrication technique. The fabricated MEAs are electrochemically characterized and the stability of MEAs is also confirmed by impedance monitoring and neural recording from cultured dissociated neurons for 3 weeks. Overall, the fabricated MEAs are comparable to the commercial MEAs. However, there is a limitation that the insulation of an SU-8 MEA deteriorates after autoclavation more rapidly than that of conventional MEAs with the SiNx insulation.

## 2. Materials and Methods

### 2.1. Microelectrode Fabrication

**Figure 1:**
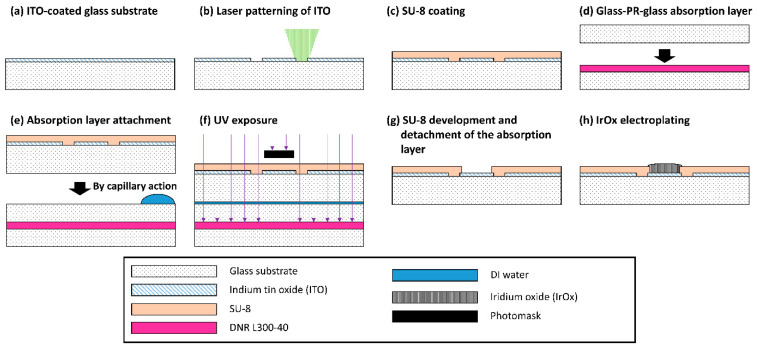
Fabrication process of a MEA with laser-patterned ITO and SU-8 insulation: (**a**) ITO-coated glass substrate; (**b**) laser patterning on an ITO glass substrate; (**c**) SU-8 spin coating on the patterned ITO; (**d**) DNR L300-40 photoresist spin coating on a glass wafer and attachment of another glass wafer on the spin-coated glass wafer; (**e**) attachment of the SU8-coated substrate to the absorption layer using water capillary action; (**f**) exposure of the SU-8 layer; (**g**) development of the SU-8 layer to create openings of the electrode sites and the contact pads; (**h**) IrO_X_ electroplating on the electrode sites.

**Figure 2:**
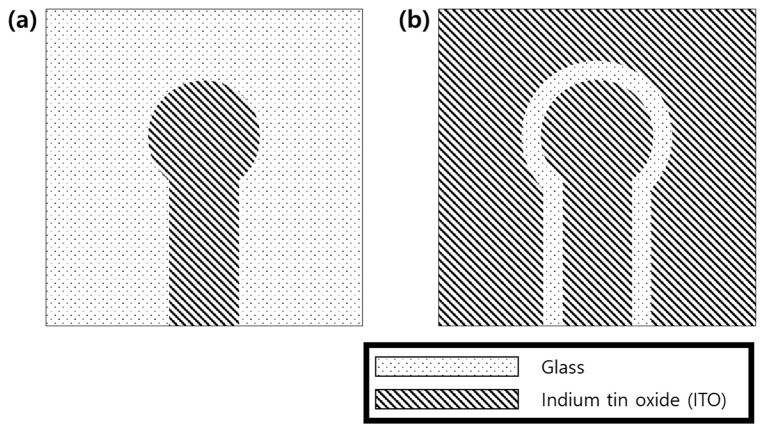
Two strategies for patterning the conductor of the microelectrode: (**a**) conventional patterning and (**b**) a conductor patterned via laser ablation of outlines along the shape of the electrode.

MEA fabrication consists of two steps including ITO metal patterning and SU-8 insulation. The overall fabrication steps are depicted in Figure 1. First, ITO is deposited via sputtering with 185 nm thickness and 1.85 × 10^−4^ Ω⋅cm resistivity on the glass (300 mm × 200 mm × 0.7 mm) by UID KOREA (Sejong, Korea). ITO-coated glass is cut into a square shape (49 mm × 49 mm) and cleaned with acetone in sonication. The ITO layer is patterned via laser ablation to form 60 channel microelectrode arrays. During laser ablation of ITO, only outlines of the conductor patterns are ablated without removing the ITO layer in the background area as shown in Figure 2b to reduce the fabrication time. The outlines of conductors are repeatedly exposed to 50 kHz pulsed UV laser six times with a power of 0.247 W and a mark speed of 200 mm/s (ProtoLaser U3, LPKF laser & Electronics AG, Garbsen, Germany; TODOC Co., Ltd. (Seoul, Korea)).

On the patterned conductors, the SU-8 negative photoresist (PR) (SU-8 2, MicroChem, Westborough, MA, USA) is spin-coated (1.3 μm thickness) at 3000 rpm for 30 s. The photoresist is then soft-baked on a hot plate for 90 s at 65 °C and for 90 s at 95 °C consecutively. In order to prevent diffuse reflection from the bottom during UV exposure, a glass–PR–glass absorption layer is created using a negative PR (DNR-L300-40 (12cP), DONGJIN SEMICHEM Co., Ltd., Seoul, Korea) as shown in Figure 1d [30]. The negative PR is spin-coated at 3000 rpm for 35 s on a 4-inch glass wafer, and another 4-inch glass wafer is placed on the PR-coated glass wafer. The glass–PR–glass absorption layer is attached under the SU-8-coated ITO glass substrate by capillary force of a drop of deionized water (Figure 1e). To expose the ITO electrode sites and contact pads, SU-8 is exposed to ultraviolet (UV) light at 150 mJ/cm^2^ using a mask aligner (MA6, SÜSS Micro Tec SE, Garching, Germany). After UV exposure, the patterned ITO glass is removed from the absorption layer (Figure 1g). After the post-exposure bake (PEB) with the same parameters as the soft bake step, the exposed SU-8 insulation layer is developed with an SU-8 developer (MicroChem, Westborough, MA, USA) for 60 s, rinsed with isopropyl alcohol (IPA), and dried with nitrogen gas. The photolithography process is completed with a curing process (hard bake) at 150 °C for 1800 s. Finally, a plastic ring is attached with PDMS to form a chamber for electrophysiological experiments.

### 2.2. Iridium Oxide Electroplating

In order to decrease the electrode impedance, the ITO electrodes are electroplated with iridium oxide (IrO_X_) in an aqueous solution of 4 mM IrCl_4_ and the supporting electrolytes of 40 mM oxalic acid and 340 mM K_2_CO_3_ modified from the previous studies [31,32]. To make the electroplating solution, IrCl_4_ was dissolved in deionized water for 2 h followed by addition of oxalic acid. Then, dissolved K_2_CO_3_ in deionized water was slowly added to make the final pH of 10.3. This solution was allowed to sit for 48 h before the electroplating.

**Figure 3:**
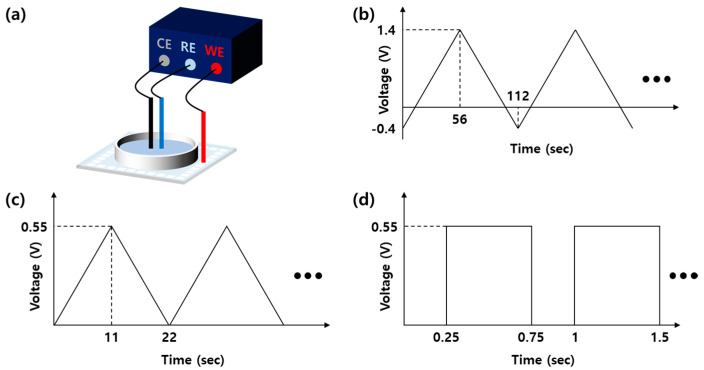
Iridium oxide electroplating of a MEA. (**a**) A three-electrode system configuration with a potentiostat; (**b**) triangular waveforms for initial electrode cleaning; (**c**) triangular and (**d**) rectangular pulses for IrO_X_ electroplating.

A three-electrode system was used for the electroplating process consisting of a Ag/AgCl reference electrode (MF-2052, Bioanalytical Systems, Inc., West Lafayette, IN, USA), a platinum wire as a counter electrode (CHI115, CH Instruments, Inc., Bee Cave, TX, USA), and the ITO electrodes of the fabricated MEA for a working electrode as shown in Figure 3a. The potential was controlled with a potentiostat (ZIVE SP2, WonATech Co., Ltd., Seoul, Korea). All of the ITO electrodes were electroplated at the same time. Prior to the electroplating process, the particles on the ITO electrode surface were removed by sweeping the voltage three times from −0.4 V to 1.4 V at a rate of 50 mV/s with 1 M of a sulfuric acid solution (Figure 3b). Iridium oxide was electroplated using triangular waveforms followed by rectangular pulses. For the first step of electroplating, 100 triangular waveforms were applied (0.0 V and 0.55 V sweeping) versus the Ag/AgCl reference electrode at the 50 mV/s sweep rate with an electroplating solution (Figure 3c). Immediately after the triangular sweeping, rectangular pulses were applied at 1 Hz with a pulse duration of 0.5 s at 0.55 V (Figure 3d). In order to find the optimized condition, different numbers of rectangular pulses (1800, 3600, and 5400) were tested. The total time taken to electroplate one MEA ranged from 1 h 30 m to 2 h 30 m depending on the number of rectangular pulses. After the electroplating, the electroplated surface was investigated using a field-emission scanning electron microscope (FE-SEM) (S-4800, Hitachi, Ltd., Tokyo, Japan).

### 2.3. Electrochemical Measurements

In order to characterize the electrochemical properties of the fabricated electrodes, electrochemical impedance spectroscopy (EIS) was conducted using the potentiostat in phosphate-buffered saline (PBS) (Life Technologies Corp., Grand Island, NY, USA). The amplitude of the sinusoidal perturbation voltage was 10 mV_pp_, and the impedance was measured in the frequency range from 100 Hz to 100 kHz. Cyclic voltammetry (CV) was also conducted by sweeping the electrochemical potential between −0.6 V and 0.8 V versus an Ag/AgCl reference electrode at the 50 mV/s sweep rate. From the obtained CV curve, cathodic charge storage capacity (CSC_C_) was calculated using the time integral of the cathodic current in the CV graph.

Since MEAs are often recycled in the laboratory, the stability of MEAs is very crucial, especially for insulation characteristics. Therefore, the immediate effect of cleaning processes and the long-term effect of exposure to the culture condition on the electrode impedance were investigated. First, the electrochemical impedance of the fabricated microelectrode was measured before and after two different cleaning processes of (i) autoclavation (121 °C, 12.5 atm, 15 min) and (ii) cleaning in a rocking shaker filled with 70% ethanol for 15 min, followed by ultraviolet (UV) light overnight for 10 h. Second, in order to monitor the insulation property during exposure to culture conditions, the impedance was repeatedly measured while the MEAs were placed in the culture environment in a 5% CO_2_/95% air humidified atmosphere at 37 °C after filling with a culture medium containing the Neurobasal medium (Life Technologies Corp.), 2% B27 (Life Technologies Corp.), and 1% Glutamax (Life Technologies Corp.) for up to 3 weeks. When measuring the electrode impedance, the culture medium was replaced with PBS. The impedance was measured every day for the first week and every two to three days for the following weeks. To prevent the increase in the medium osmolality due to water evaporation, the volume of the medium was maintained by refilling distilled water. As a control group, impedance monitoring was also performed with commercial MEAs with the SiN_X_ insulation (60MEA200/30iR-ITO-pr, Multi Channel Systems MCS GmbH, Reutlingen, Germany).

### 2.4. Cell Culture

Primary hippocampal neurons were obtained from fetuses of Sprague–Dawley (SD) rats (Samtako, Osan, Korea) at embryonic day 18. Hippocampi were isolated from brains of fetuses and placed in a cold Hanks’ balanced salt solution (HBSS) (Life Technologies Corp., Grand Island, NY, USA). The dissected hippocampi were digested in 0.5% Trypsin-EDTA (Life Technologies Corp.) for 15 min at 37 °C. The digested hippocampi were rinsed five times in PBS and triturated gently with a pipette. The cells were seeded at a density of 1800 cells/mm^2^ on the MEA coated with a poly-L-lysine solution (P4707, SIGMA-ALDRICH, Co., St. Louis, MO, USA) in a plating medium containing Dulbecco’s modified Eagle’s medium (DMEM) (Life Technologies Corp.), 10% horse serum (Life Technologies Corp.), and 1% anti-anti (Life Technologies Corp.). After incubation for 30 min, the plating medium were replaced with the Neurobasal medium (Life Technologies Corp.) supplemented with 2% B27 (Life Technologies Corp.) and 1% Glutamax (Life Technologies Corp.). The cultures were incubated in a 5% CO_2_/95% air humidified atmosphere at 37 °C. Half of the medium was replaced with a fresh medium twice a week. The SiN_X_-insulated MEAs (Multi Channel Systems MCS GmbH) were also used for neural culture as a control condition. All animal procedures were approved by the Institutional Animal Care and Use Committee (IACUC) at Ewha Womans University (IACUC 20-034). Images of cultured neurons on the MEAs were obtained with an inverted microscope (IX71, Olympus, Tokyo, Japan) using a CMOS camera (Zyla 5.5 sCMOS, Andor Technology Ltd., Belfast, UK).

### 2.5. Electrophysiological Recording of Neurons

The electrophysiological recording was performed at 10, 17, and 21 days in vitro (DIV) from the neurons cultured on both the fabricated MEAs and the commercial SiNx-insulated MEAs. The recordings were performed using a signal amplifier (MEA 1060-Inv-BC, Multi Channel Systems MCS GmbH, Reutlingen, Germany) and a data acquisition device (USB-ME64-System, Multi Channel Systems MCS GmbH, Reutlingen, Germany) connected to a data acquisition computer. During the recording, the temperature was maintained at 37 °C with a temperature controller (TC02, Multi Channel Systems MCS GmbH, Reutlingen, Germany). The recorded data were processed with the MC_Rack software (version 4.6.2, Multi Channel Systems MCS GmbH, Reutlingen, Germany). The signals were sampled at the frequency of 25 kHz and filtered using a second-order Butterworth high-pass filter with the cutoff frequency of 200 Hz. For each recording session, the spontaneous activity from cultured neurons was measured for 1 min.

From the recorded neural signals, individual spikes were detected with the amplitude threshold. The threshold level was automatically determined as 5× standard deviation which was calculated from the background noise recorded for 0.5 s. From the detected spikes, in addition, neuronal bursting patterns were analyzed. Bursts were detected based on the Max Interval algorithm [33]. The parameters used for burst detection were as follows: (1) maximum inter-spike interval to initiate a burst: 10 ms, (2) minimum number of spikes in a burst: five, (3) minimum duration of a burst: 50 ms, and (4) minimum inter-spike interval between two bursts: 210 ms.

### 2.6. Statistical Analysis

All the values were provided as the means ± the standard error of the mean (SEM). Statistical analysis was performed using an unpaired *t*-test (Mann–Whitney test) using Prism 9 (version 9.0.0 (121), GraphPad Software, San Diego, CA, USA, 2020); *p*-values less than 0.05 were considered statistically significant.

## 3. Results

### 3.1. MEA Fabrication

**Figure 4:**
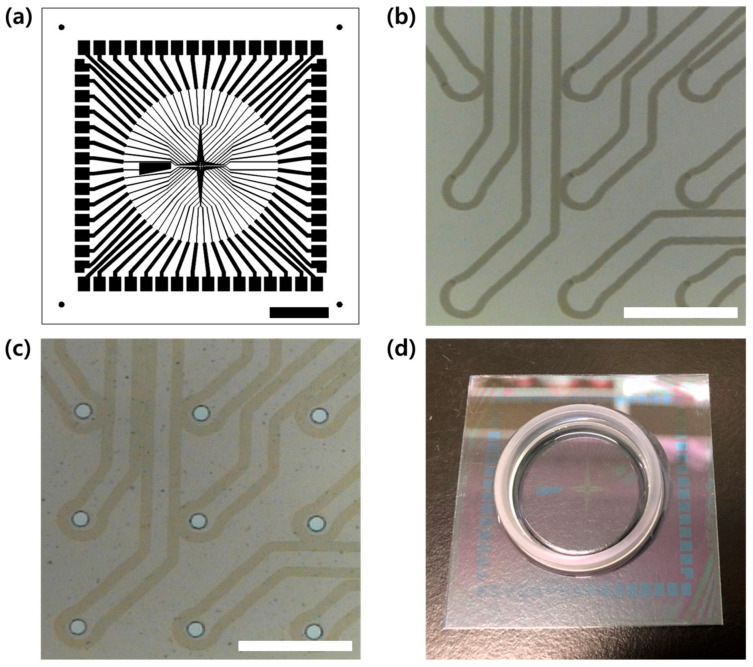
Fabrication of a MEA with laser-patterned ITO and SU-8 insulation. (**a**) Overview of the MEA conductor layout, scale bar = 10 mm. (**b**) Laser-patterned conductor outlines, scale bar = 200 μm. (**c**) Exposed electrode sites after SU-8 insulation, scale bar = 200 μm. (**d**) Fabricated MEA attached to a plastic ring.

Sixty-channel MEAs were successfully fabricated via laser patterning of the ITO conductor and SU-8 insulation as shown in Figure 4. Laser exposure successfully eliminated the thin-film ITO along the outlines of conductor patterns with the line width of approximately 15 μm (Figure 4b). At the speed of 200 mm/s, the laser patterning process took less than 60 s. After laser patterning, the step height profile was measured as 207 nm using a surface profiler (DektakXT-A, Bruker Corp., Billerica, MA, USA), which is more than the thickness of the ITO layer (185 nm). A short circuit test with a multimeter also confirmed that the background and conductor regions were electrically isolated.

The patterned ITO electrodes were insulated with SU-8, and the electrode sites were successfully opened via the photolithography process (Figure 4c). Therefore, unlike in the conventional methods, there was no need for several steps of masking and etching. The diameter of the site-opened electrode was 30 μm.

### 3.2. Electrochemical Characterization

**Figure 5:**
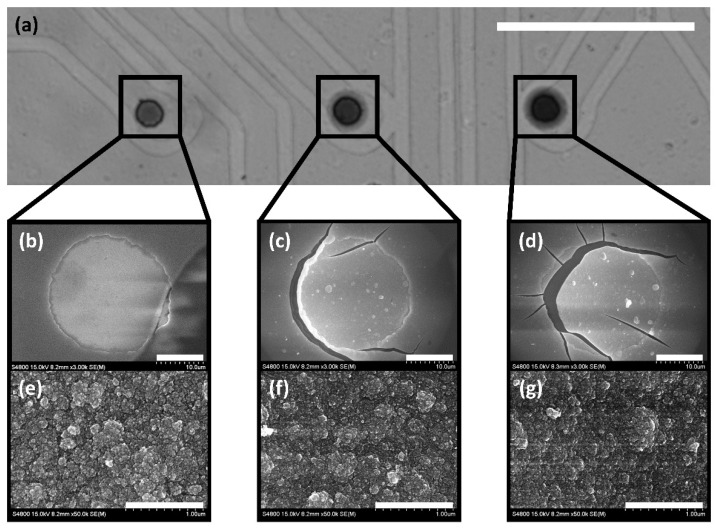
Iridium oxide electroplating of a MEA. Different numbers of rectangular voltage pulses were applied to each microelectrode (left electrode: 1800 times, middle electrode: 3600 times, right electrode: 5400 times). (**a**) Widefield microscope image of the IrO_X_ electroplated microelectrodes, scale bar = 200 μm. (**b**–**d**) FE-SEM images of the IrO_X_ electroplated microelectrodes, scale bar = 10 μm. (**e**–**g**) FE-SEM images of the IrO_X_ electroplated surfaces, scale bar = 1 μm.

To decrease the impedance of the microelectrode and increase the charge storage capacity (CSC), iridium oxide (IrO_X_) was electroplated on the ITO surface of electrodes. After electroplating with 1800, 3600, and 5400 rectangular pulses, the magnitudes of impedances at 1 kHz were 42.80 ± 4.13 kΩ, 66.59 ± 8.13 kΩ, and 50.96 ± 12.45 kΩ, and the values of CSC were 67.73 ± 7.69 mC/cm^2^, 71.27 ± 22.59 mC/cm^2^, and 73.28 ± 27.24 mC/cm^2^, respectively (*n* = 6 for each condition). The measured impedance and CSC of the IrO_X_ electrodes were similar to the values in the existing literature [32,34,35]. Based on these results, electroplating with 1800 rectangular pulses showed the best electrochemical properties because it resulted in the lowest impedance magnitude while the CSC was similar to that at other conditions. Even though the increased number of rectangular pulses resulted in larger and darker electrode sites in a widefield microscope image as shown in Figure 5a, the FE-SEM images showed no significant difference in surface morphology (Figure 5e–g). However, as the number of rectangular pulses increased, the larger cracking of IrO_X_ was observed (Figure 5b–d). Therefore, electroplating of the fabricated MEAs was performed with 1800 rectangular pulses.

**Figure 6:**
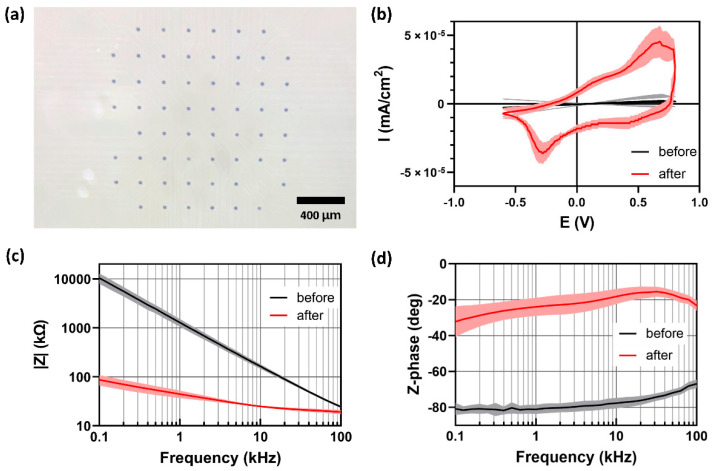
IrO_X_ electroplating and electrochemical characterization. (**a**) IrO_X_-electroplated electrode array, (**b**) CV curves, (**c**) magnitudes and (**d**) phase of impedance before and after the electroplating. The mean and the standard deviation are shown in each graph.

The electrochemical properties of the fabricated MEAs were compared before and after the iridium oxide electroplating with 1800 rectangular voltage pulses. As shown in Figure 6a, the electrode sites became dark due to the formation of porous IrO_X_. After the electroplating, the CSC, the time integral of the cyclic voltammogram in Figure 6b, increased more than 48 times from 0.70 mC/cm^2^ to 62.81 mC/cm^2^. Electrochemical impedance spectroscopy results also dramatically changed, as shown in Figure 6c. The impedance magnitude at 1 kHz decreased by more than 40 times from 1.72 MΩ to 42.03 kΩ. The impedance phase graphs showed that the electrode–electrolyte impedance became more resistive due to the faradaic reactions in IrO_X_.

**Figure 7:**
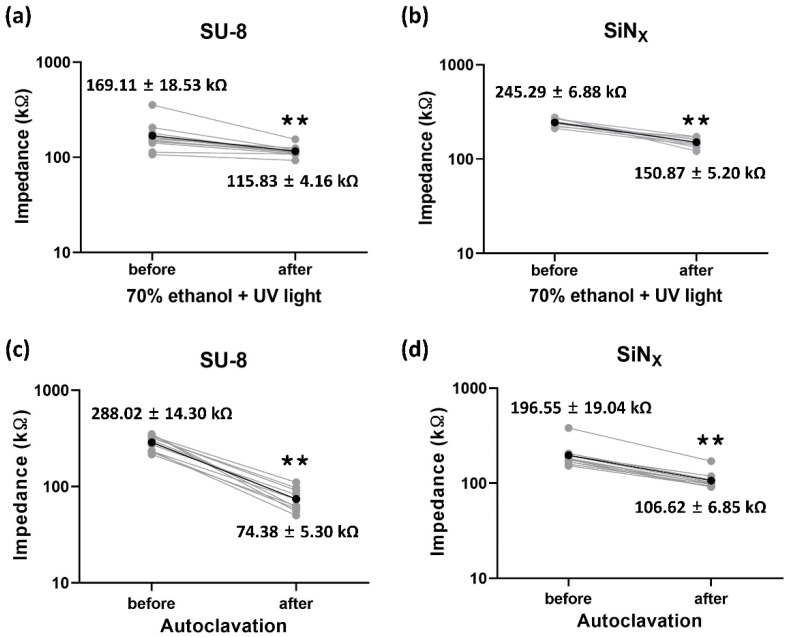
Impedance change after the MEA cleaning processes. Impedance change of (**a**) the fabricated SU-8-insulated MEA and (**b**) the SiN_X_-insulated MEA before and after cleaning with 70% ethanol and UV. Impedance change of (**c**) the fabricated SU-8-insulated MEA and (**d**) the SiN_X_-insulated MEA before and after autoclave cleaning. The gray dots and lines are individual measured values and the black dots and lines are the average values (** *p* < 0.01).

**Figure 8:**
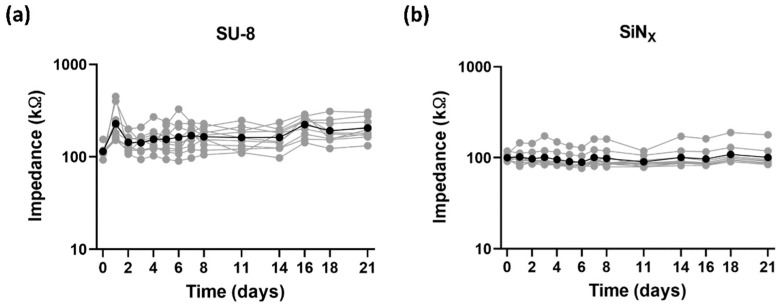
Long-term impedance monitoring in culture-like environments. (**a**) Impedance magnitude changes of the fabricated SU-8-insulated MEAs and (**b**) the SiN_X_-insulated MEAs. The grays are individual measured values and the blacks are the average values.

Figure 7 shows the impedance change induced by the MEA cleaning processes. Cleaning with 70% ethanol and UV light caused the impedance changes from 169.11 ± 18.53 kΩ to 115.83 ± 4.16 kΩ (*n* = 12) and from 245.29 ± 6.88 kΩ to 150.87 ± 5.20 kΩ (*n* = 10) for the SU-8- and SiN_X_-insulated MEAs, respectively. Both MEAs showed similar significant decreases in impedance magnitude. After autoclave cleaning, however, the impedance of the SU-8 MEAs (change from 288.02 ± 14.30 kΩ to 74.38 ± 5.30 kΩ, *n* = 12) decreased more dramatically than that of the SiN_X_ MEAs (change from 196.55 ± 19.04 kΩ to 106.62 ± 6.85 kΩ, *n* = 11). Although the cleaning processes affect the impedance, long-term exposure to culture conditions did not significantly change the impedance in both SU-8 (from 114.49 ± 4.91 kΩ at day 0 to 205.44 ± 16.97 kΩ at day 21, *n* = 10) and SiN_X_ (from 100.20 ± 2.62 kΩ at day 0 to 100.80 ± 9.23 kΩ at day 21, *n* = 10) MEAs, as shown in Figure 8. The impedance of the fabricated SU-8 MEAs were relatively unstable compared with the SiN_X_ MEAs but not significantly decreased for 21 days.

### 3.3. Electrophysiology

**Figure 9:**
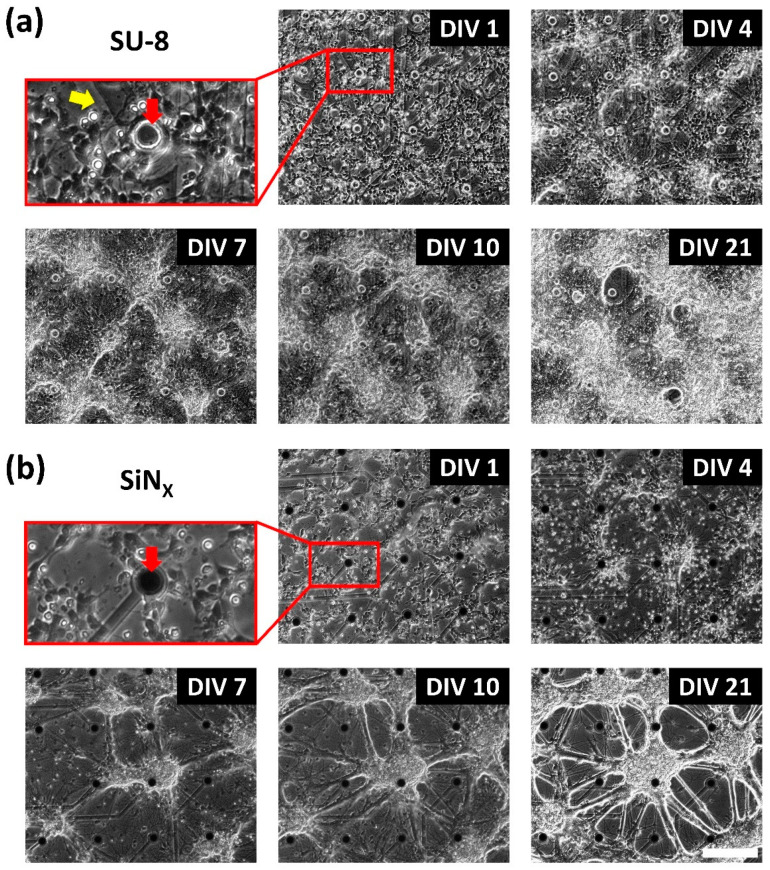
Hippocampal neurons cultured on MEAs. The growth of hippocampal neurons cultured for 21 days on (**a**) an SU-8-insulated MEA by microfabrication and (**b**) on a SiN_X_-insulated MEA as a control condition. It is confirmed that the proposed MEAs with laser-patterned ITO and SU-8 insulation are suitable for long-term cultures. The red arrows indicate each electrode site and the yellow arrow indicates the laser-ablated area. Scale bar = 200 μm.

Figure 9 shows the morphology of hippocampal neurons cultured for 21 days on the SU-8 MEAs and the SiN_X_-insulated MEAs as a control condition. The hippocampal neurons were well-attached to the MEA SU-8 surface at DIV 1. As the hippocampal neurons grew over time, the connections between neurons were formed and strengthened over time in similar manners for both MEAs. Due to the high seeding density of 1800 cells/mm^2^, the aggregation of neuronal cells occurred in both conditions after DIV 7, but the SU-8 MEAs showed relatively uniform spatial distributions.

**Figure 10:**
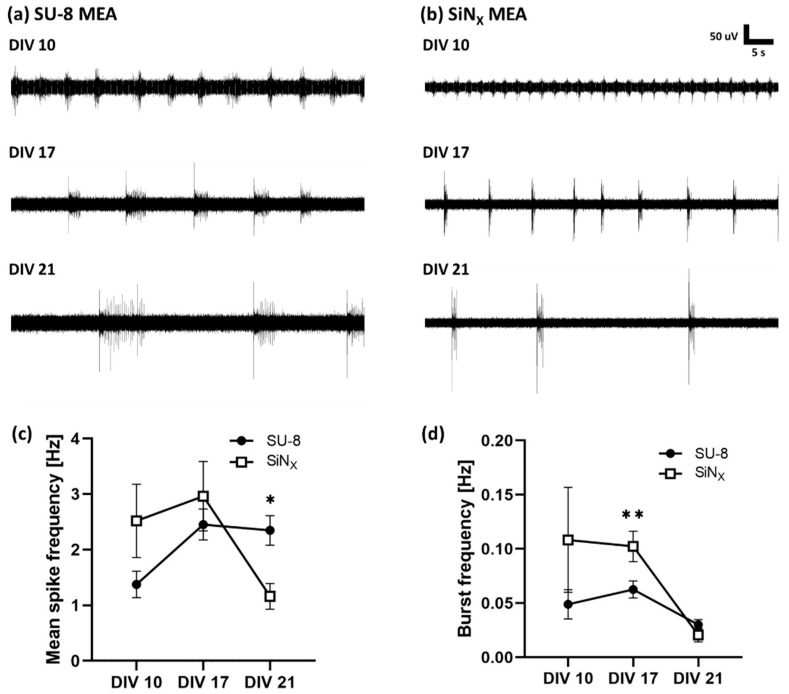
Spontaneous neural activity recorded from hippocampal neurons via MEAs. The waveforms recorded from neurons cultured on (**a**) the fabricated MEAs with SU-8 and laser-patterned ITO and (**b**) the SiN_X_-insulated MEAs as a control group. (**c**) Mean spike frequencies and (**d**) burst frequencies of spontaneous neural activity from cultured neurons on the fabricated MEAs and on the SiN_X_ MEAs (* *p* < 0.05, ** *p* < 0.01).

At different timepoints of DIV 10, 17, and 21, the spontaneous neural activities were recorded from the cultured neurons using the fabricated MEAs and the SiN_X_-insulated MEAs (Figure 10). The results showed that the amplitude of the spike signal gradually increased, as shown in Figure 10a,b. The noise level (peak-to-peak voltage of the background waveform) was ±17~18 μV for the MEAs insulated with SU-8 and ±12~13 μV for the MEAs insulated with SiN_X_. The mean spike frequency from the SU-8 MEAs between DIV 10 and DIV 17 was lower than that from the SiN_X_ MEAs (Figure 10c) but higher at DIV 21. It indicates that maturation of the neural network is slower on the fabricated SU-8 MEAs, but is maintained for a longer period. Burst frequency of the recorded neural signals from the SU-8 MEAs was considerably smaller than that from the SiN_X_ MEAs at DIV 10 and DIV 17 (Figure 10d).

## 4. Discussion and Conclusions

In this study, a new method of MEA fabrication is proposed using a laser-patterned ITO conductor and SU-8 insulation. Compared with the conventional methods for MEA fabrication, it is a simple and low-cost process, which could be advantageous for large-scale experiments with a number of MEAs. Typical microfabrication process for conductor patterning of MEAs comprises metal deposition, photolithography (photoresist coating, baking, exposure, development, etc.), etching or lift-off process, and several washing, rinsing, and drying steps. However, the laser ablation process took only a minute to pattern all ITO conductor lines of a 60-channel electrode array. SU-8 insulation can also reduce the cost and time for MEA fabrication because it is just a single photolithography process while conventional methods require deposition of the insulation layer, photolithography, etching to expose electrode sites, and cleaning steps.

In addition, laser fabrication enables easy customization of the MEA designs such as electrode size or arrangement. It is another advantage of the proposed method because different designs of MEAs are frequently needed for the experiments with different cells or tissues. Without an additional cost for a new photomask, laser fabrication can change the design of MEAs simply by changing the CAD file for laser control. Even though the proposed method employed SU-8 photolithography with a photomask in this study, an alternative method can be applied with polymer insulation and laser patterning of the polymer. Previously, we showed that a laser can pattern various thin-film polymers as well as metals such as polydimethylsiloxane (PDMS), liquid crystal polymer, and thin film of platinum and the platinum–iridium alloy [36,37]. Eventually, therefore, MEAs can be fabricated without any high-cost thin-film microfabrication processes such as photolithography, etching, and wet processes. However, in terms of patterning resolution, it is obvious that the thin-film process with photolithography is superior to laser patterning because photolithography can conventionally make submicron patterns while laser patterning is limited by the spot size of at least ten micrometers.

As an electrode site material, iridium oxide (IrO_X_) was electroplated on the exposed ITO surfaces in this study. Since there exist several different oxidation states of iridium, it is well-known that IrO_X_ provides a higher charge injection limit and a lower impedance than other electrode materials such as platinum and titanium nitride [38]. As shown in Figure 6, after IrO_X_ electroplating, both the impedance magnitude at 1 kHz and the charge storage capacity were improved approximately 40 times compared with the bare ITO surface. Due to its high charge injection limit, IrO_X_ are commonly used for stimulating electrode materials. It is also well-known that its degradation issues for chronic use should be further investigated [39]. However, the results of 3-week impedance monitoring showed that IrO_X_-electroplated electrodes are not degraded (Figure 8) and may be used for neural recording (Figure 9). Although IrO_X_ electrodes are verified only for 3 weeks, the degradation and stability issue will not be problematic because they are used only for detection of neural activity without any active charge transfer reaction for stimulation.

SU-8 is an epoxy-based negative photoresist that is commonly used in microelectromechanical systems (MEMS) for soft lithography or imprinting techniques [40,41]. Since SU-8 is proven to be biocompatible and chemically stable, it is used as a material of microfluidics for life science applications. Recently, SU-8 has also been used for the substrate or insulation of microelectrode arrays [42,43,44]. Even though the biocompatibility of SU-8 is proven, a subtle change of the culture environment can dramatically affect the viability of cultured neurons and the long-term stability cannot be guaranteed in the harsh culture environment. Therefore, impedance monitoring tests were performed to verify the stability of SU-8 insulation and the primary neurons were cultured on the MEAs. The long-term impedance monitoring showed that SU-8 is chemically stable in the culture conditions without any significant change for 3 weeks (Figure 8). The cleaning process with ethanol and UV induced approximately 30% decrease in impedance magnitude, but it was not significantly different from the SiN_X_-insulated MEAs, as shown in Figure 7. However, autoclavation (121 °C, 12.5 atm, 15 min) resulted in a 75% decrease in impedance magnitude, which implies that the high temperature and pressure of autoclavation deteriorate the insulation property of the SU-8 material. It is because SU-8 has a higher water absorption rate and is thus more vulnerable to the increased temperature and pressure than SiN_X_ [45,46,47]. In the aspect of insulation, SiN_X_ has a superior insulation property because it has a higher dielectric constant, around 6~7, while the dielectric constant of SU-8 is known to be around 3. It might explain the higher noise level in the SU-8 insulation than in the SiN_X_ one, as shown in Figure 10. However, the noise level of SU-8 MEAs is also sufficiently low to detect neural activity.

The dissociated neurons from rat embryo hippocampi were cultured on the fabricated MEAs and extracellular neural recording was performed in order to verify the feasibility and the biocompatibility of the MEAs. As shown in Figure 9, the rat hippocampal neurons grew for 3 weeks without any harmful effects. From the cultured neurons, the spontaneous activity was observed from DIV 10 (Figure 10). The spike frequencies from the cultured neurons on the fabricated MEAs were higher at DIV 21 compared with the commercial MEAs with the SiNx insulation. However, the burst frequencies were lower at DIV 10 and DIV 17. Since the aggregated neurons can produce a higher burst activity, it is consistent with the result in Figure 9 showing that the cultured neurons on the SiN_X_ surface aggregated more than on the SU-8 surface. As long as less aggregation and uniform distribution of cultured neurons imply that a surface is more suitable for neuronal attachment, the results show that SU-8 MEAs can maintain long-term stable neuronal cultures.

The main challenges of the proposed MEA fabrication are mainly related with the processes for transparent materials. First, laser patterning of ITO on the glass substrate should be carefully performed because both materials are transparent. The laser wavelength and power should be appropriate for the target materials [48]. In this study, a 50 kHz pulsed UV laser (355 nm) was employed to eliminate ITO and the repetition and speed of the laser were precisely adjusted. Therefore, the laser wavelength and the parameters need to be properly chosen for different materials in consideration of its absorbance spectrum. Second, there is an issue in SU-8 photolithography which should be noted because the use of transparent substrates can make the photolithography process difficult due to reflection and scattering lights during UV exposure. Especially, the precision of the negative photoresist pattern can be easily affected by the reflected light from the chuck on the bottom, the diffracted light from the edge of mask patterns, or the scattered light. In this study, therefore, an additional structure was adopted for precise patterning of the SU-8 photoresist because the lithography of the SU-8 insulation layer was performed on a transparent glass substrate and transparent ITO layers. First, according to the previous study, we attempted to use a silicon wafer as a backing layer during UV exposure to eliminate the effect of irregular light reflection from the chuck [30]. Although the precision of SU-8 patterns was improved with the Si backing layer, the electrode sites with 30 μm were not completely formed due to the overexposed edge region. It is likely that the reflected light from the Si wafer is diffracted at the edge of the photomask patterns. Therefore, as described in this study, the glass–photoresist–glass absorption layer was employed instead of a Si wafer because the photoresist in the backing layer absorbs UV light and prevents light reflection. As a result, this strategy effectively eliminates the problem and results in precise SU-8 patterns for the insulation layer of MEAs. As a future work, the method described in this study can be improved to fabricate MEAs only with laser micromachining, without photolithography. As discussed above, various insulating materials and metals can be patterned by laser. Since the technology for laser micromachining is rapidly developing, laser patterning could substitute thin-film processes with a relatively low resolution. In addition, fabricated MEAs should be further tested for the extended period and for recycling in order to systematically investigate the failure modes of MEAs.
